# Di-μ-methano­lato-κ^4^ *O*:*O*-bis­[bis­(3-methyl-5-phenyl-1*H*-pyrazole-κ*N* ^2^)(nitrato-κ*O*)copper(II)]

**DOI:** 10.1107/S160053681201241X

**Published:** 2012-03-31

**Authors:** Xu-Guang Li, Meng-Meng Gao, Seik Weng Ng

**Affiliations:** a Fundamental Laboratory of Life Science, Medical School of Tibet University for Nationalities, Xianyang 712082, People’s Republic of China; b School of Materials Science and Engineering, Beijing Institute of Technology, Beijing 100081, People’s Republic of China; c Department of Chemistry, University of Malaya, 50603 Kuala Lumpur, Malaysia, and Chemistry Department, King Abdulaziz University, PO Box 80203 Jeddah, Saudi Arabia

## Abstract

Copper nitrate in methanol solution cleaves the N—C_methanol_ bond when reacted with 3-methyl-5-phenyl­pyrazole-1-methanol to yield the centrosymmetric dinuclear title compound, [Cu_2_(CH_3_O)_2_(NO_3_)_2_(C_10_H_10_N_2_)_4_], in which the Cu^II^ atom is linked to a nitrate ion, two methano­late ions and two pyrazole ligands in a distorted square-pyramidal environment. The O atom of the nitrate anion occupies the apical site. The crystal structure features intra­molecular N—H⋯O hydrogen bonds.

## Related literature

For a related structure, see: He & Sykes (2007 ▶). For the synthesis of 3-methyl-5-phenyl­pyrazole-1-methanol, see: Zhu *et al.* (2004 ▶).
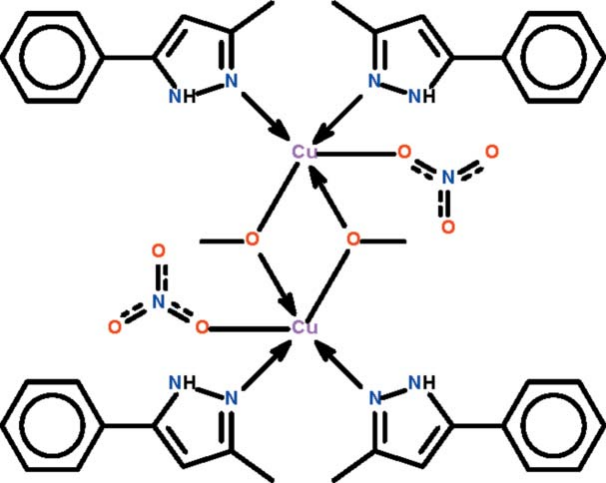


## Experimental

### 

#### Crystal data

[Cu_2_(CH_3_O)_2_(NO_3_)_2_(C_10_H_10_N_2_)_4_]
*M* *_r_* = 945.97
Triclinic, 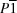

*a* = 8.3896 (8) Å
*b* = 11.2569 (11) Å
*c* = 12.7200 (12) Å
α = 106.120 (2)°
β = 103.025 (2)°
γ = 95.853 (2)°
*V* = 1106.85 (18) Å^3^
*Z* = 1
Mo *K*α radiation
μ = 1.02 mm^−1^
*T* = 293 K
0.12 × 0.11 × 0.10 mm

#### Data collection

Bruker SMART-1000 diffractometer
Absorption correction: multi-scan (*SADABS*; Sheldrick, 1996 ▶) *T*_min_ = 0.887, *T*_max_ = 0.905
6768 measured reflections
4898 independent reflections
3461 reflections with *I* > 2σ(*I*)
*R*_int_ = 0.015

#### Refinement

*R*[*F* ^2^ > 2σ(*F* ^2^)] = 0.042
*wR*(*F* ^2^) = 0.116
*S* = 1.02
4898 reflections
290 parameters
2 restraints
H atoms treated by a mixture of independent and constrained refinement
Δρ_max_ = 0.38 e Å^−3^
Δρ_min_ = −0.30 e Å^−3^

### 

Data collection: *SMART* (Bruker, 2001 ▶); cell refinement: *SAINT* (Bruker, 2001 ▶); data reduction: *SAINT*; program(s) used to solve structure: *SHELXS97* (Sheldrick, 2008 ▶); program(s) used to refine structure: *SHELXL97* (Sheldrick, 2008 ▶); molecular graphics: *X-SEED* (Barbour, 2001 ▶); software used to prepare material for publication: *publCIF* (Westrip, 2010 ▶).

## Supplementary Material

Crystal structure: contains datablock(s) global, I. DOI: 10.1107/S160053681201241X/xu5488sup1.cif

Structure factors: contains datablock(s) I. DOI: 10.1107/S160053681201241X/xu5488Isup2.hkl

Additional supplementary materials:  crystallographic information; 3D view; checkCIF report

## Figures and Tables

**Table 1:** Hydrogen-bond geometry (Å, °)

*D*—H⋯*A*	*D*—H	H⋯*A*	*D*⋯*A*	*D*—H⋯*A*
N2—H2⋯O2^i^	0.87 (1)	2.26 (2)	3.012 (3)	144 (3)
N4—H4⋯O3	0.87 (1)	2.17 (2)	3.022 (4)	166 (3)

Symmetry code: (i) 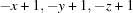.

**Fig. 1.**
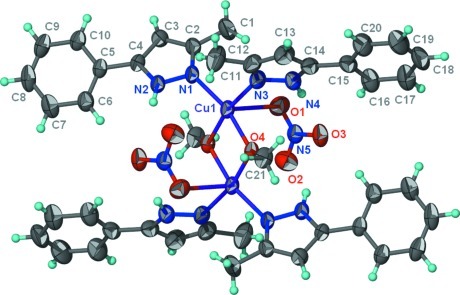
Thermal ellipsoid plot (Barbour, 2001) of centrosymmetric [Cu(OCH3)(NO3)(C10H10N2)2]2 at the 50% probability level; hydrogen atoms are drawn as spheres of arbitrary radius. Inversion-related atoms are not labeled.

## supplementary crystallographic information

### Comment

Copper nitrate in methanol solution cleaves the *N*–C_methanol_ bond when reacted with 3-methyl-5-phenylpyrazole-1-methanol to yield the dinuclear title compound (Scheme I, Fig. 1). The molecule lies on a center-of-inversion; the Cu_II_ atom is linked to a nitrate ion, two methanolate ions and two of the pyrazole ligands in a square-pyramidal environment. In the perchlorate analog, [Cu(OCH_3_)(C_10_H_10_N_2_)_2_]_2_(ClO_4_)_2_,the counterion is not connected to the copper atom, whose geometry is a square pyramid. The compound was synthesized by directly reacting 3-methyl-5-phenylpyrazole with copper perchlorate in methanol medium (He & Sykes, 2007).

### Experimental

3-Methyl-5-phenylpyrazole-1-methanol was synthesized by using a literature procedure (Zhu *et al.*, 2004). The ligand (0.065 g, 0.4 mmol) was dissolved in dichloromethane (10 mol) and this was mixed with a methanol solution (10 ml) of copper nitrate trihydrate (0.024 g, 0.1 mmol). The clear blue solution was filtered and then set aside for the growth of deep blue crystals. CH&N elemental analysis. Calc. for C_42_H_46_Cu_2_N_10_O_8_: C 53.33, H 4.90; N 14.81%. Found: C 56.36, H 5.18, N 15.02%.

### Refinement

Carbon-bound H-atoms were placed in calculated positions (C—H 0.93 to 0.96 Å) and were included in the refinement in the riding model approximation, with *U*(H) set to 1.2 to 1.5*U*(C).

The amino H-atoms were located in a difference Fourier map, and were refined with a distance restraint of N–H 0.88±0.01 Å; their temperature factors were freely refined.

### Crystal data

**Table:**

[Cu_2_(CH_3_O)_2_(NO_3_)_2_(C_10_H_10_N_2_)_4_]	*Z* = 1
*M**_r_* = 945.97	*F*(000) = 490
Triclinic, *P*1	*D*_x_ = 1.419 Mg m^−^^3^
Hall symbol: -P 1	Mo *K*α radiation, λ = 0.71073 Å
*a* = 8.3896 (8) Å	Cell parameters from 2170 reflections
*b* = 11.2569 (11) Å	θ = 2.5–24.5°
*c* = 12.7200 (12) Å	µ = 1.02 mm^−^^1^
α = 106.120 (2)°	*T* = 293 K
β = 103.025 (2)°	Prism, blue
γ = 95.853 (2)°	0.12 × 0.11 × 0.10 mm
*V* = 1106.85 (18) Å^3^	

### Data collection

**Table:**

Bruker SMART-1000 diffractometer	4898 independent reflections
Radiation source: fine-focus sealed tube	3461 reflections with *I* > 2σ(*I*)
Graphite monochromator	*R*_int_ = 0.015
ω scans	θ_max_ = 27.5°, θ_min_ = 1.9°
Absorption correction: multi-scan (*SADABS*; Sheldrick, 1996)	*h* = −10→10
*T*_min_ = 0.887, *T*_max_ = 0.905	*k* = −12→14
6768 measured reflections	*l* = −16→10

### Refinement

**Table:**

Refinement on *F*^2^	Primary atom site location: structure-invariant direct methods
Least-squares matrix: full	Secondary atom site location: difference Fourier map
*R*[*F*^2^ > 2σ(*F*^2^)] = 0.042	Hydrogen site location: inferred from neighbouring sites
*wR*(*F*^2^) = 0.116	H atoms treated by a mixture of independent and constrained refinement
*S* = 1.02	*w* = 1/[σ^2^(*F*_o_^2^) + (0.0516*P*)^2^ + 0.4958*P*] where *P* = (*F*_o_^2^ + 2*F*_c_^2^)/3
4898 reflections	(Δ/σ)_max_ = 0.001
290 parameters	Δρ_max_ = 0.38 e Å^−^^3^
2 restraints	Δρ_min_ = −0.30 e Å^−^^3^

### Fractional atomic coordinates and isotropic or equivalent isotropic displacement parameters (Å^2^)

**Table:**

	*x*	*y*	*z*	*U*_iso_*/*U*_eq_	
Cu1	0.54841 (4)	0.37278 (3)	0.47584 (3)	0.04912 (14)	
O1	0.7401 (3)	0.4733 (3)	0.3606 (2)	0.0769 (7)	
O2	0.6684 (3)	0.6571 (2)	0.3847 (2)	0.0768 (7)	
O3	0.5511 (3)	0.5059 (2)	0.23096 (17)	0.0703 (6)	
O4	0.3871 (2)	0.46286 (18)	0.41469 (14)	0.0464 (5)	
N1	0.7022 (3)	0.3017 (2)	0.57615 (18)	0.0508 (6)	
N2	0.6938 (3)	0.3252 (2)	0.68576 (19)	0.0507 (6)	
N3	0.4722 (3)	0.2251 (2)	0.3377 (2)	0.0576 (6)	
N4	0.4708 (3)	0.2394 (3)	0.2345 (2)	0.0573 (6)	
N5	0.6566 (3)	0.5464 (3)	0.3264 (2)	0.0535 (6)	
C1	0.9035 (5)	0.2356 (4)	0.4677 (3)	0.0793 (11)	
H1A	0.8149	0.2370	0.4056	0.119*	
H1B	0.9330	0.1535	0.4525	0.119*	
H1C	0.9983	0.2965	0.4764	0.119*	
C2	0.8479 (4)	0.2663 (3)	0.5748 (2)	0.0519 (7)	
C3	0.9314 (4)	0.2661 (3)	0.6822 (2)	0.0545 (7)	
H3	1.0350	0.2440	0.7028	0.065*	
C4	0.8307 (3)	0.3052 (3)	0.7523 (2)	0.0469 (6)	
C5	0.8519 (3)	0.3269 (3)	0.8744 (2)	0.0493 (7)	
C6	0.7739 (4)	0.4139 (3)	0.9353 (3)	0.0603 (8)	
H6	0.7085	0.4603	0.8990	0.072*	
C7	0.7932 (4)	0.4319 (4)	1.0499 (3)	0.0730 (10)	
H7	0.7408	0.4906	1.0901	0.088*	
C8	0.8892 (4)	0.3639 (4)	1.1049 (3)	0.0753 (11)	
H8	0.8993	0.3748	1.1815	0.090*	
C9	0.9694 (4)	0.2801 (4)	1.0461 (3)	0.0717 (10)	
H9	1.0362	0.2353	1.0835	0.086*	
C10	0.9524 (4)	0.2610 (3)	0.9315 (3)	0.0579 (8)	
H10	1.0082	0.2040	0.8925	0.069*	
C11	0.3473 (6)	0.0666 (4)	0.4104 (3)	0.0996 (15)	
H11A	0.4399	0.1010	0.4763	0.149*	
H11B	0.3330	−0.0234	0.3870	0.149*	
H11C	0.2484	0.0925	0.4281	0.149*	
C12	0.3794 (5)	0.1132 (3)	0.3157 (3)	0.0659 (9)	
C13	0.3201 (5)	0.0571 (3)	0.1989 (3)	0.0731 (10)	
H13	0.2534	−0.0214	0.1618	0.088*	
C14	0.3786 (4)	0.1393 (3)	0.1488 (3)	0.0582 (8)	
C15	0.3536 (4)	0.1299 (3)	0.0279 (3)	0.0616 (8)	
C16	0.4062 (5)	0.2286 (4)	−0.0078 (3)	0.0764 (10)	
H16	0.4597	0.3052	0.0456	0.092*	
C17	0.3799 (5)	0.2146 (5)	−0.1229 (3)	0.0884 (12)	
H17	0.4166	0.2815	−0.1461	0.106*	
C18	0.3000 (6)	0.1023 (5)	−0.2022 (3)	0.0935 (14)	
H18	0.2828	0.0925	−0.2792	0.112*	
C19	0.2461 (7)	0.0050 (5)	−0.1673 (3)	0.1131 (18)	
H19	0.1906	−0.0708	−0.2212	0.136*	
C20	0.2722 (6)	0.0169 (4)	−0.0533 (3)	0.0944 (14)	
H20	0.2353	−0.0507	−0.0310	0.113*	
C21	0.2225 (4)	0.4090 (3)	0.3499 (3)	0.0620 (8)	
H21A	0.1667	0.4727	0.3290	0.093*	
H21B	0.1647	0.3730	0.3940	0.093*	
H21C	0.2241	0.3446	0.2825	0.093*	
H2	0.602 (2)	0.349 (3)	0.698 (3)	0.061 (10)*	
H4	0.510 (4)	0.3130 (18)	0.231 (3)	0.079 (12)*	

### Atomic displacement parameters (Å^2^)

**Table:**

	*U*^11^	*U*^22^	*U*^33^	*U*^12^	*U*^13^	*U*^23^
Cu1	0.0482 (2)	0.0578 (2)	0.03230 (18)	0.00578 (16)	−0.00269 (13)	0.01163 (15)
O1	0.0655 (15)	0.0950 (19)	0.0817 (17)	0.0317 (14)	0.0191 (13)	0.0398 (15)
O2	0.0714 (16)	0.0622 (15)	0.0842 (17)	0.0014 (13)	0.0201 (13)	0.0070 (14)
O3	0.0657 (14)	0.0978 (18)	0.0423 (12)	0.0083 (13)	0.0082 (10)	0.0205 (12)
O4	0.0393 (10)	0.0574 (12)	0.0334 (9)	0.0009 (9)	−0.0025 (8)	0.0123 (9)
N1	0.0506 (14)	0.0663 (16)	0.0343 (11)	0.0126 (12)	0.0056 (10)	0.0174 (11)
N2	0.0439 (14)	0.0703 (17)	0.0409 (12)	0.0157 (13)	0.0089 (11)	0.0217 (12)
N3	0.0634 (16)	0.0554 (16)	0.0418 (13)	0.0061 (13)	−0.0030 (11)	0.0109 (12)
N4	0.0658 (17)	0.0528 (16)	0.0400 (13)	0.0032 (13)	0.0029 (12)	0.0047 (12)
N5	0.0435 (14)	0.0709 (18)	0.0501 (14)	0.0076 (13)	0.0165 (11)	0.0225 (14)
C1	0.074 (2)	0.114 (3)	0.0451 (18)	0.029 (2)	0.0173 (17)	0.011 (2)
C2	0.0500 (17)	0.0613 (19)	0.0406 (15)	0.0102 (14)	0.0093 (13)	0.0118 (14)
C3	0.0439 (16)	0.068 (2)	0.0475 (16)	0.0148 (15)	0.0051 (13)	0.0148 (15)
C4	0.0457 (15)	0.0516 (17)	0.0411 (14)	0.0053 (13)	0.0041 (12)	0.0180 (13)
C5	0.0423 (15)	0.0620 (19)	0.0402 (14)	0.0003 (13)	0.0032 (12)	0.0199 (14)
C6	0.0504 (18)	0.081 (2)	0.0470 (16)	0.0099 (16)	0.0088 (14)	0.0196 (16)
C7	0.053 (2)	0.107 (3)	0.0500 (18)	0.0045 (19)	0.0137 (15)	0.0134 (19)
C8	0.056 (2)	0.119 (3)	0.0397 (16)	−0.012 (2)	0.0029 (15)	0.025 (2)
C9	0.060 (2)	0.095 (3)	0.0545 (19)	0.0006 (19)	−0.0066 (16)	0.037 (2)
C10	0.0499 (17)	0.073 (2)	0.0492 (16)	0.0066 (15)	0.0039 (13)	0.0259 (16)
C11	0.140 (4)	0.077 (3)	0.071 (2)	−0.007 (3)	0.002 (3)	0.035 (2)
C12	0.078 (2)	0.0510 (19)	0.0567 (19)	0.0079 (17)	−0.0037 (17)	0.0158 (16)
C13	0.085 (3)	0.0482 (19)	0.062 (2)	−0.0004 (18)	−0.0088 (18)	0.0060 (16)
C14	0.0610 (19)	0.0508 (18)	0.0482 (17)	0.0117 (15)	−0.0006 (14)	0.0035 (14)
C15	0.061 (2)	0.064 (2)	0.0430 (16)	0.0170 (16)	−0.0007 (14)	−0.0006 (15)
C16	0.073 (2)	0.088 (3)	0.0516 (19)	−0.003 (2)	0.0066 (17)	0.0086 (19)
C17	0.078 (3)	0.122 (4)	0.059 (2)	0.008 (3)	0.017 (2)	0.024 (2)
C18	0.098 (3)	0.125 (4)	0.045 (2)	0.033 (3)	0.015 (2)	0.006 (2)
C19	0.162 (5)	0.090 (3)	0.048 (2)	0.022 (3)	0.000 (3)	−0.017 (2)
C20	0.138 (4)	0.065 (2)	0.052 (2)	0.007 (2)	0.003 (2)	−0.0041 (18)
C21	0.0456 (17)	0.071 (2)	0.0514 (17)	−0.0038 (15)	−0.0093 (13)	0.0134 (16)

### Geometric parameters (Å, º)

**Table:**

Cu1—O4^i^	1.9185 (19)	C7—C8	1.377 (5)
Cu1—O4	1.9256 (18)	C7—H7	0.9300
Cu1—N3	1.979 (2)	C8—C9	1.366 (5)
Cu1—N1	1.992 (2)	C8—H8	0.9300
Cu1—Cu1^i^	2.9939 (8)	C9—C10	1.385 (4)
O1—N5	1.239 (3)	C9—H9	0.9300
O2—N5	1.242 (3)	C10—H10	0.9300
O3—N5	1.261 (3)	C11—C12	1.505 (5)
O4—C21	1.412 (3)	C11—H11A	0.9600
O4—Cu1^i^	1.9185 (19)	C11—H11B	0.9600
N1—C2	1.326 (4)	C11—H11C	0.9600
N1—N2	1.364 (3)	C12—C13	1.391 (4)
N2—C4	1.344 (3)	C13—C14	1.370 (5)
N2—H2	0.873 (10)	C13—H13	0.9300
N3—C12	1.333 (4)	C14—C15	1.478 (4)
N3—N4	1.363 (3)	C15—C16	1.380 (5)
N4—C14	1.348 (4)	C15—C20	1.388 (5)
N4—H4	0.874 (10)	C16—C17	1.392 (5)
C1—C2	1.501 (4)	C16—H16	0.9300
C1—H1A	0.9600	C17—C18	1.370 (6)
C1—H1B	0.9600	C17—H17	0.9300
C1—H1C	0.9600	C18—C19	1.365 (6)
C2—C3	1.388 (4)	C18—H18	0.9300
C3—C4	1.379 (4)	C19—C20	1.383 (6)
C3—H3	0.9300	C19—H19	0.9300
C4—C5	1.469 (4)	C20—H20	0.9300
C5—C6	1.387 (4)	C21—H21A	0.9600
C5—C10	1.393 (4)	C21—H21B	0.9600
C6—C7	1.383 (4)	C21—H21C	0.9600
C6—H6	0.9300		
			
O4^i^—Cu1—O4	77.69 (8)	C8—C7—H7	119.6
O4^i^—Cu1—N3	166.83 (9)	C6—C7—H7	119.6
O4—Cu1—N3	91.96 (9)	C9—C8—C7	119.4 (3)
O4^i^—Cu1—N1	91.67 (9)	C9—C8—H8	120.3
O4—Cu1—N1	165.30 (9)	C7—C8—H8	120.3
N3—Cu1—N1	99.88 (10)	C8—C9—C10	120.8 (3)
O4^i^—Cu1—Cu1^i^	38.93 (5)	C8—C9—H9	119.6
O4—Cu1—Cu1^i^	38.76 (5)	C10—C9—H9	119.6
N3—Cu1—Cu1^i^	130.23 (7)	C5—C10—C9	120.1 (3)
N1—Cu1—Cu1^i^	129.86 (7)	C5—C10—H10	119.9
C21—O4—Cu1^i^	124.42 (19)	C9—C10—H10	119.9
C21—O4—Cu1	125.07 (19)	C12—C11—H11A	109.5
Cu1^i^—O4—Cu1	102.31 (8)	C12—C11—H11B	109.5
C2—N1—N2	105.4 (2)	H11A—C11—H11B	109.5
C2—N1—Cu1	133.5 (2)	C12—C11—H11C	109.5
N2—N1—Cu1	117.77 (18)	H11A—C11—H11C	109.5
C4—N2—N1	111.9 (2)	H11B—C11—H11C	109.5
C4—N2—H2	133 (2)	N3—C12—C13	109.7 (3)
N1—N2—H2	115 (2)	N3—C12—C11	120.9 (3)
C12—N3—N4	105.7 (2)	C13—C12—C11	129.3 (3)
C12—N3—Cu1	132.4 (2)	C14—C13—C12	107.1 (3)
N4—N3—Cu1	119.6 (2)	C14—C13—H13	126.5
C14—N4—N3	111.5 (3)	C12—C13—H13	126.5
C14—N4—H4	128 (2)	N4—C14—C13	106.0 (3)
N3—N4—H4	119 (2)	N4—C14—C15	123.3 (3)
O1—N5—O2	122.3 (3)	C13—C14—C15	130.7 (3)
O1—N5—O3	119.0 (3)	C16—C15—C20	118.7 (3)
O2—N5—O3	118.6 (3)	C16—C15—C14	123.0 (3)
C2—C1—H1A	109.5	C20—C15—C14	118.3 (3)
C2—C1—H1B	109.5	C15—C16—C17	120.6 (4)
H1A—C1—H1B	109.5	C15—C16—H16	119.7
C2—C1—H1C	109.5	C17—C16—H16	119.7
H1A—C1—H1C	109.5	C18—C17—C16	120.0 (4)
H1B—C1—H1C	109.5	C18—C17—H17	120.0
N1—C2—C3	110.3 (3)	C16—C17—H17	120.0
N1—C2—C1	120.8 (3)	C19—C18—C17	119.5 (4)
C3—C2—C1	128.8 (3)	C19—C18—H18	120.3
C4—C3—C2	106.6 (3)	C17—C18—H18	120.3
C4—C3—H3	126.7	C18—C19—C20	121.3 (4)
C2—C3—H3	126.7	C18—C19—H19	119.4
N2—C4—C3	105.7 (2)	C20—C19—H19	119.4
N2—C4—C5	121.9 (3)	C19—C20—C15	119.8 (4)
C3—C4—C5	132.4 (3)	C19—C20—H20	120.1
C6—C5—C10	118.7 (3)	C15—C20—H20	120.1
C6—C5—C4	120.9 (3)	O4—C21—H21A	109.5
C10—C5—C4	120.4 (3)	O4—C21—H21B	109.5
C7—C6—C5	120.2 (3)	H21A—C21—H21B	109.5
C7—C6—H6	119.9	O4—C21—H21C	109.5
C5—C6—H6	119.9	H21A—C21—H21C	109.5
C8—C7—C6	120.7 (4)	H21B—C21—H21C	109.5
			
O4^i^—Cu1—O4—C21	−149.3 (3)	C2—C3—C4—C5	−178.2 (3)
N3—Cu1—O4—C21	38.9 (2)	N2—C4—C5—C6	−27.0 (4)
N1—Cu1—O4—C21	−104.9 (4)	C3—C4—C5—C6	151.7 (3)
Cu1^i^—Cu1—O4—C21	−149.3 (3)	N2—C4—C5—C10	153.5 (3)
O4^i^—Cu1—O4—Cu1^i^	0.0	C3—C4—C5—C10	−27.7 (5)
N3—Cu1—O4—Cu1^i^	−171.78 (10)	C10—C5—C6—C7	−1.5 (5)
N1—Cu1—O4—Cu1^i^	44.5 (4)	C4—C5—C6—C7	179.0 (3)
O4^i^—Cu1—N1—C2	−104.9 (3)	C5—C6—C7—C8	−0.2 (5)
O4—Cu1—N1—C2	−148.1 (3)	C6—C7—C8—C9	1.7 (5)
N3—Cu1—N1—C2	68.7 (3)	C7—C8—C9—C10	−1.4 (5)
Cu1^i^—Cu1—N1—C2	−113.3 (3)	C6—C5—C10—C9	1.8 (5)
O4^i^—Cu1—N1—N2	50.9 (2)	C4—C5—C10—C9	−178.7 (3)
O4—Cu1—N1—N2	7.7 (5)	C8—C9—C10—C5	−0.4 (5)
N3—Cu1—N1—N2	−135.5 (2)	N4—N3—C12—C13	0.2 (4)
Cu1^i^—Cu1—N1—N2	42.5 (2)	Cu1—N3—C12—C13	162.2 (3)
C2—N1—N2—C4	−0.1 (3)	N4—N3—C12—C11	−177.8 (3)
Cu1—N1—N2—C4	−162.1 (2)	Cu1—N3—C12—C11	−15.7 (5)
O4^i^—Cu1—N3—C12	−141.7 (4)	N3—C12—C13—C14	−0.5 (4)
O4—Cu1—N3—C12	−103.9 (3)	C11—C12—C13—C14	177.2 (4)
N1—Cu1—N3—C12	67.3 (3)	N3—N4—C14—C13	−0.6 (4)
Cu1^i^—Cu1—N3—C12	−110.6 (3)	N3—N4—C14—C15	179.3 (3)
O4^i^—Cu1—N3—N4	18.4 (6)	C12—C13—C14—N4	0.7 (4)
O4—Cu1—N3—N4	56.2 (2)	C12—C13—C14—C15	−179.2 (3)
N1—Cu1—N3—N4	−132.6 (2)	N4—C14—C15—C16	−7.8 (5)
Cu1^i^—Cu1—N3—N4	49.4 (3)	C13—C14—C15—C16	172.1 (4)
C12—N3—N4—C14	0.3 (4)	N4—C14—C15—C20	172.8 (3)
Cu1—N3—N4—C14	−164.5 (2)	C13—C14—C15—C20	−7.3 (6)
N2—N1—C2—C3	0.5 (3)	C20—C15—C16—C17	−0.8 (6)
Cu1—N1—C2—C3	158.4 (2)	C14—C15—C16—C17	179.8 (3)
N2—N1—C2—C1	−178.3 (3)	C15—C16—C17—C18	0.5 (6)
Cu1—N1—C2—C1	−20.5 (5)	C16—C17—C18—C19	0.3 (7)
N1—C2—C3—C4	−0.8 (4)	C17—C18—C19—C20	−0.8 (8)
C1—C2—C3—C4	178.0 (3)	C18—C19—C20—C15	0.5 (8)
N1—N2—C4—C3	−0.4 (3)	C16—C15—C20—C19	0.3 (7)
N1—N2—C4—C5	178.6 (3)	C14—C15—C20—C19	179.7 (4)
C2—C3—C4—N2	0.7 (3)		

Symmetry code: (i) −*x*+1, −*y*+1, −*z*+1.

### Hydrogen-bond geometry (Å, º)

**Table:**

*D*—H···*A*	*D*—H	H···*A*	*D*···*A*	*D*—H···*A*
N2—H2···O2^i^	0.87 (1)	2.26 (2)	3.012 (3)	144 (3)
N4—H4···O3	0.87 (1)	2.17 (2)	3.022 (4)	166 (3)

Symmetry code: (i) −*x*+1, −*y*+1, −*z*+1.
